# Emergency Presentation of Invasive Cervical Cancer in Late Pregnancy: A Case Report and Literature Review

**DOI:** 10.7759/cureus.46900

**Published:** 2023-10-12

**Authors:** Ali H Ali, Sara E Marhoon, Mohamed Taman

**Affiliations:** 1 College of Medicine, Mansoura University, Mansoura, EGY; 2 Department of Obstetrics and Gynecology, Mansoura University, Mansoura, EGY

**Keywords:** obstetric emergency, gynecologic malignancy, cervical cancer, case report, late pregnancy, antepartum hemorrhage

## Abstract

Antepartum hemorrhage (APH) often prompts consideration of the presence of obstetric disorders. Here, we describe a case with active APH in which invasive cervical cancer was the cause. A 41-year-old woman, fifth gravida, fourth para (G5, P4), presented to the emergency department at 38 weeks of gestation with an acute severe attack of vaginal bleeding, which occurred immediately after a per-vaginal examination at another local institute. Despite initial stabilization measures and investigations to exclude common causes of APH, a protruding cervical mass was discovered during a Cusco speculum examination. The patient underwent an emergent cesarean section (CS). Postoperatively, the patient was referred to the gynecological oncology unit for further evaluation and management. Magnetic resonance imaging (MRI) confirmed the presence of a large cervical mass. A punch biopsy revealed squamous cell carcinoma (SCC) of the cervix. All these confirmed the condition as cervical carcinoma stage IB3. This case and literature review highlight the obstacles that might delay the diagnosis of cervical cancer and the importance of continuing the screening program strategies even during pregnancy to avoid complications of invasive cervical cancer. In addition, bleeding due to cervical cancer should always be considered one of the important differential diagnoses of APH even in full-term pregnancy.

## Introduction

Antepartum hemorrhage (APH) is a serious obstetric complication that endangers both the mother and the fetus, increasing morbidity and mortality rates. It accounts for between 0.5% and 5% of all obstetrical emergencies [1]. APH has a wide range of causes, including common etiologies such as placenta previa, abruptio placenta, and vasa previa, as well as uncommon causes such as genital trauma, infections, varicose veins, and urogenital malignancies [1].

Cervical cancer is known to be the most common invasive genital tract malignancy during pregnancy [2]. Nonetheless, cervical cancer is a complicated disease during pregnancy since the vast majority of individuals are asymptomatic. If symptoms do appear, they are frequently vague, such as vaginal discharge, abdominal pain, and irregular vaginal bleeding, which might be mistakenly attributed to typical physiological changes during pregnancy [3]. As a result, a comprehensive history and physical examination must be done to detect uncommon causes of APH. Cervical cancer should be kept in the differential diagnosis of vaginal bleeding during pregnancy as it has a real challenge in management, and many factors play a major role in the decision-making process, such as the tumor stage, gestational age, and fetal lung maturity [3].

In this context, we will present a case of APH due to an invasive cervical tumor. This case demonstrates cervical cancer as a cause of APH and the obstacles that may delay its management, emphasizing the significance of early and accurate diagnosis to optimize patient outcomes.

## Case presentation

A 41-year-old woman, fifth gravida, fourth para (G5, P4), at 38 weeks of gestation, presented to the emergency department due to an acute severe attack of vaginal bleeding that started one hour before the admission. The bleeding was provoked by a per-vaginal examination at another non-governmental medical facility. It was significant, characterized by a fresh red color and the absence of noticeable clots.

The initial stabilization and anti-shock measures were taken after the insertion of two large-bore cannulas for intravenous access, and blood samples were drawn for various tests such as complete blood count (CBC), blood grouping, and cross-matching. Two and a half liters of crystalloid and broad-spectrum antibiotics were started immediately. Subsequently, an ultrasound was performed to rule out common conditions such as placenta previa and placental abruption and to confirm fetal well-being by assessing the biophysical profile. The ultrasound revealed that the placenta was normally situated, and no retroplacental hematoma was detected.

After stabilizing the patient’s general condition, a comprehensive medical history was obtained to gain clarity on her case. The patient was married with four children and reported having her first sexual intercourse at the age of 18. Moreover, her marriage is monogamous. She had irregularly used combined oral contraceptive pills, and two intrauterine devices were inserted between pregnancies. The patient had experienced two instances of postcoital bleeding one year before her current pregnancy. Additionally, she complained of recurring, untreated white, foul-smelling vaginal discharge, accompanied by dull lower abdominal pain. The patient had never undergone a Pap smear test. Unfortunately, episodes of vaginal bleeding persisted throughout her pregnancy, leading her to seek non-governmental medical advice during her irregular antenatal care visits, as she was uncommitted to the antenatal care visits, which are provided by the Egyptian Ministry of Health in primary health care units. Regrettably, the treating obstetrician prescribed nonspecific hemostatic measures without further investigation, resulting in only partial relief. The patient's past medical and surgical history revealed no notable events. Based on her ongoing complaints, at the 38-week gestational follow-up appointment, her obstetrician did a local examination that resulted in vaginal bleeding, so the obstetrician referred her to our emergency department. During her evaluation, a Cusco speculum examination was performed, revealing a protruding cervical mass measuring approximately 5x5 cm. After starting the stabilization measures, informed consent from the patient and her husband was obtained for an emergent cesarean section (CS), which resulted in the birth of a healthy male baby weighing 3200 g. After the end of the delivery, we provisionally diagnosed this condition as cervical cancer, and thus a punch biopsy was taken from the cervical mass. Following the surgery, the patient was admitted to the gynecological oncology unit for further assessment and management.

**Figure 1:**
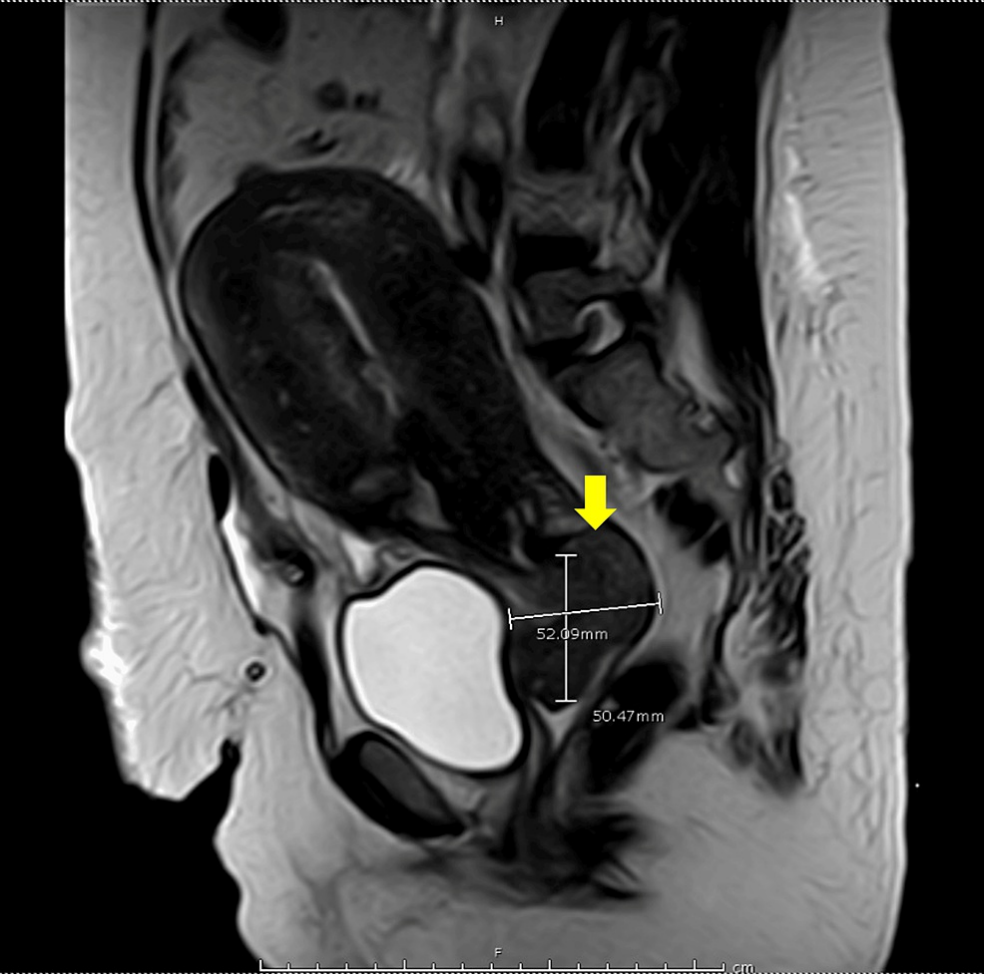
Abdominopelvic T2-weighted MRI showing enhanced soft tissue cervical mass measuring 5.2x5 cm. MRI: magnetic resonance imaging.

Initial laboratory investigations revealed anemia (8.5 g/dL), hypoalbuminemia (2.5 g/dL), and a urinary tract infection with a significant number of pus cells (>100/HPF), red blood cells (>100/HPF), and abundant bacteria. Other results were normal. Investigations were conducted to determine the nature of the cervical mass. Magnetic resonance imaging (MRI) revealed a sizable, well-defined, enhanced soft tissue mass with high signal intensity on T2-weighted images. This mass extended from the cervix into the upper vagina and had contact with the anterior aspect of the urinary bladder. The mass also exerted pressure on the surrounding rectum and rectosigmoid colon, possibly infiltrating these areas. Its dimensions were 5.2x5 cm (Figure 1).

**Figure 2:**
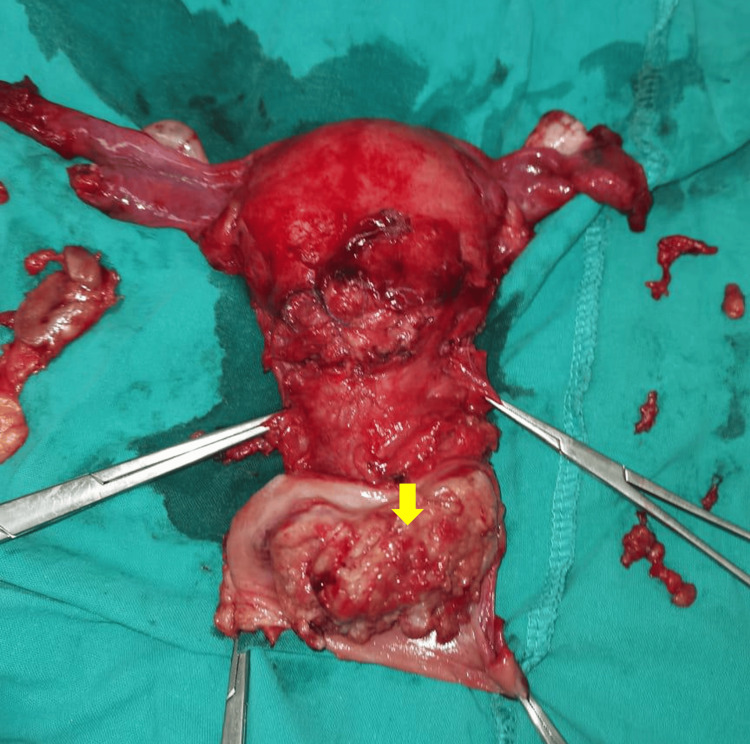
Postoperative specimen. The arrow indicates the location of the cervical tumor.

A metastatic evaluation was conducted, involving cystoscopy and colonoscopy, both of which yielded negative results. Moreover, an examination under anesthesia to evaluate the extension of the lesion and the presence of parametrial infiltration revealed a cauliflower-like cervical mass involving both the upper and lower cervical lips without infiltrating the parametrium. The biopsy confirmed the presence of cervical squamous cell carcinoma (SCC) at stage IB3. Following a comprehensive multidisciplinary discussion and patient counseling, a Wertheim's operation was performed (Figure 2). Pathological analysis of the resected mass confirmed the presence of SCC, as it revealed malignant proliferation in the form of epithelial cell sheets and nests with a high degree of atypia, pleomorphism, minimal keratinization, frequent mitosis, and lymphovascular emboli. The radical margins of the specimen and the parametrium were free, while the upper part of the vagina was infiltrated. One out of two lymph nodes was infiltrated by the tumor tissue. The patient was subsequently referred to the clinical oncology and nuclear medicine departments for further evaluation of the need for adjuvant chemoradiotherapy.

## Discussion

We retrieved eight similar cases of APH as a first presentation for invasive cervical cancer in the literature, which are summarized in Table 1 [4-9]. Most of the patients were diagnosed around the age of 30. The youngest reported case was 25 years [7], while the oldest was 37 years [9]. In our case, the patient was 41 years. Only one of the eight cases was primigravida at the time of diagnosis [5]. All the cases were diagnosed through cervical biopsies or surgical procedures. The stages of invasive cervical cancer varied from IB1 to IIIC1. In all cases, a CS and hysterectomy were performed. Furthermore, five cases received chemoradiotherapy [4,7,9], one case received chemotherapy [5], and one case received adjuvant radiotherapy [8]. Unfortunately, the patient with the worst prognosis died within 11 months following the surgery [5].

**Table 1:** Summary of the cases of invasive cervical cancer with APH as a first presentation and their clinical course from diagnosis to treatment. APH: antepartum hemorrhage; G: gravida; P: para; CS: cesarean section; SCC: squamous cell carcinoma; NACT: neoadjuvant chemotherapy; PET: positron emission tomography; NICU: neonatal intensive care unit.

Case	Patient age (year)	Gravidity and parity	Gestational age at diagnosis (weeks)	Gestational age at delivery (weeks)	Mode of delivery	Stage of the disease	Histopathologic diagnosis	Surgical procedures	Chemoradiotherapy	Postoperative follow-up	Fetal outcome
Case 1 [4]	33	G3, P2	33	34	CS	IIIC1	SCC	Hysterotomy, bilateral salpingectomy, bilateral pelvic and para-aortic lymphadenectomy, and bilateral ovarian transposition	Post-delivery chemoradiotherapy for metastatic disease	The PET scan revealed no evidence of disseminated disease	NICU female (2720 g)
Case 2 [5]	27	G1, P0	34	34	Emergent CS	III	Small cell neuroendocrine carcinoma	Myomectomy and transvaginal and abdominal hysterectomy	Chemotherapy	The patient died 11 months postoperatively	Premature male (1980 g)
Case 3 [6]	34	G6, P5	23	33	CS	IB2	SCC	Cesarean–radical hysterectomy and bilateral pelvic lymphadenectomy	-	Not mentioned	NICU Female (2128 g)
Case 4 [7]	25	G2, P1	32	32+5	Elective CS	IIA	Small cell carcinoma	Radical hysterectomy and bilateral pelvic lymphadenectomy	Chemoradiotherapy for metastatic disease	Not mentioned	NICU male
Case 5 [8]	36	G2, P1	31	37+6	Elective CS	IB2	Mucinous adenocarcinoma	Radical hysterectomy and bilateral pelvic lymphadenectomy	Adjuvant pelvic radiotherapy	The patient was free two years after radiotherapy	Not mentioned
Case 6 [9]	37	G8, P3	20	35+3	Elective CS	IB1	SCC	Radical hysterectomy, bilateral salpingectomy, pelvic lymph node dissection, and para-aortic lymph node dissection	NACT and postoperative radiotherapy	No evidence of disease	Healthy male (2470 g)
Case 7 [9]	26	G4, P1	30+6	34+5	Elective CS	IB1	SCC	Radical hysterectomy, bilateral salpingectomy, pelvic lymph node dissection, and para-aortic lymph node dissection	NACT and postoperative radiotherapy	No evidence of disease	Healthy female (2330 g)
Case 8 [9]	31	G3, P1	29+4	36+4	Elective CS	IB2	SCC	Radical hysterectomy, bilateral salpingectomy, pelvic lymph node dissection, and para-aortic lymph node dissection	NACT and postoperative radiotherapy	No evidence of disease	Healthy female (2600 g)

It is rare for cervical cancer to be discovered during pregnancy, 1%-3% [3]. On the contrary, only 0.05% of all pregnancies are complicated by cervical cancer [3]. It has been hypothesized that the physiological changes during pregnancy may mask the diagnosis, encompassing hormonal alterations and other mechanisms essential for fetal growth and development, and could potentially expedite the progression of the tumor. While the emergence of invasive cervical cancer during pregnancy remains infrequent and unexpected, it underscores the significance of cervical cancer screening both before and during gestation to facilitate early detection and proper treatment [10].

The issue of delayed diagnosis of cervical cancer is of utmost importance [11]. Advanced stages of the disease carry significantly poorer prognoses and amplify the intricacy of treatment strategies [12]. However, this condition is influenced by numerous factors. On the one hand, patient-related factors such as age, socioeconomic status, educational level, and awareness play an important role [13,14]. Notably, older women are at an elevated risk compared to their younger counterparts, although some studies have indicated heightened risk in women under 25 as opposed to those over 25 [13]. Additionally, lower socioeconomic status, limited education, and inadequate awareness among women and their partners are attributed to delayed diagnosis [13,14]. On the other hand, medical history, encompassing medical conditions, obstetric background, and family history, is equally crucial [13]. Women with a history of sexually transmitted infections (STIs) are predisposed to delayed diagnosis, while those who have not undergone local examination within the past three years are also at risk. Basically, higher parity (five to nine pregnancies) appears to mitigate the risk. Otherwise, women without a family history of invasive cervical cancer are more susceptible to late-stage diagnoses [13]. Furthermore, certain factors are involved with the healthcare system itself [13,14]. The most important factor was the healthcare provider’s non-holistic approach. Patients initially seeking care at governmental hospitals have a lower risk compared to those accessing other healthcare facilities [13].

In our case, a term pregnant woman presented to our emergency department with a provoked APH. The delay in diagnosis of cervical cancer was mainly attributed to patient unawareness, the healthcare provider's role, irregular antenatal care visits, and insufficient screening programs in such a developing country. The patient was at high risk for cervical cancer, as she experienced early sexual life with prolonged use of contraception methods, recurrent untreated vaginal tract infections, and multiparity. The patient has never been included in a screening program for cervical cancer or even performed a Pap smear.

The process of screening and diagnosing cervical cancer during pregnancy revolves around the "three-step model," encompassing cervical cytology, colposcopy, and cervical biopsy [3]. Cervical cytology is the preferred initial diagnostic test for screening for cervical cancer. This test is safe for both the pregnant woman and the fetus [3]. Existing studies substantiate the reliability of cervical cytology during pregnancy, reflecting its performance in non-pregnant women [15,16]. However, conducting colposcopy during pregnancy proves challenging due to hormonal fluctuations. Hence, this procedure is most effective during the first and second trimesters. Additionally, a cervical biopsy can be accomplished through colposcopy or direct visual assessment, especially when a high-grade lesion or cancer is suspected [3]. The management of cervical cancer during pregnancy necessitates an individualized approach, primarily dependent on the disease stage and the gestational age of the patient [17].

## Conclusions

With the presence of routine cervical screening programs for early cancer detection, it is very rare to encounter patients with cervical cancer during pregnancy. To ensure consistency among physicians, it is advisable to follow a standardized algorithm for episodes of bleeding during pregnancy involving cervical cancer. Early diagnosis of this disease is essential for better outcomes and prognosis. So, it is crucial to adhere to antenatal care visits with accredited obstetricians. Additionally, it is important to promote women's reproductive and sexual health awareness alongside cervical cancer awareness and screening, even in developing countries, to enhance favorable outcomes.

## References

[1] Oguejiofor CB, Okafor CD, Eleje GU (2023). A five-year review of feto-maternal outcome of antepartum haemorrhage in a tertiary center. Int J Innov Res Med Sci.

[2] Botha MH, Rajaram S, Karunaratne K (2018). Cancer in pregnancy. Int J Gynaecol Obstet.

[3] Beharee N, Shi Z, Wu D, Wang J (2019). Diagnosis and treatment of cervical cancer in pregnant women. Cancer Med.

[4] Fox CR, Burdeaux S, Downing KT (2021). A 33-year-old woman in the third trimester of pregnancy diagnosed with advanced-staged squamous cell cervical carcinoma by magnetic resonance imaging and biopsy. Am J Case Rep.

[5] Pan L, Liu R, Sheng X, Chen D (2019). Small cell neuroendocrine carcinoma of the cervix in pregnancy: a case report and review. Case Rep Obstet Gynecol.

[6] Tewari K, Cappuccini F, Balderston KD, Rose GS, Porto M, Berman ML (1998). Pregnancy in a Jehovah's witness with cervical cancer and anemia. Gynecol Oncol.

[7] Liu H, Yang X, Zhang C, Liu X (2014). Small cell carcinoma of the cervix at 32-week gestation: a case report and review of the literature. Lab Med.

[8] Wang JC, Bernard L, Boutross-Tadross O (2022). Estrogen receptor-positive adenocarcinoma of the cervix presenting during pregnancy: two case reports and review of the literature. Gynecol Oncol Rep.

[9] Huang H, Quan Y, Qi X, Liu P (2021). Neoadjuvant chemotherapy with paclitaxel plus cisplatin before radical surgery for locally advanced cervical cancer during pregnancy: a case series and literature review. Medicine (Baltimore).

[10] Perrone AM, Bovicelli A, D'Andrilli G, Borghese G, Giordano A, De Iaco P (2019). Cervical cancer in pregnancy: analysis of the literature and innovative approaches. J Cell Physiol.

[11] Hull R, Mbele M, Makhafola T (2020). Cervical cancer in low and middle-income countries. Oncol Lett.

[12] Hui D (2015). Prognostication of survival in patients with advanced cancer: predicting the unpredictable?. Cancer Control.

[13] Allahqoli L, Dehdari T, Rahmani A (2022). Delayed cervical cancer diagnosis: a systematic review. Eur Rev Med Pharmacol Sci.

[14] Plaisy MK, Boni SP, Coffie PA (2023). Barriers to early diagnosis of cervical cancer: a mixed-method study in Côte d'Ivoire, West Africa. BMC Womens Health.

[15] Morice P, Uzan C, Gouy S, Verschraegen C, Haie-Meder C (2012). Gynaecological cancers in pregnancy. Lancet.

[16] Morimura Y, Fujimori K, Soeda S (2002). Cervical cytology during pregnancy--comparison with non-pregnant women and management of pregnant women with abnormal cytology. Fukushima J Med Sci.

[17] Amant F, Berveiller P, Boere IA (2019). Gynecologic cancers in pregnancy: guidelines based on a third international consensus meeting. Ann Oncol.

